# Reliability and Validity of the Multimedia Activity Recall in Children and Adults (MARCA) in People with Chronic Obstructive Pulmonary Disease

**DOI:** 10.1371/journal.pone.0081274

**Published:** 2013-11-27

**Authors:** Toby Hunt, Marie T. Williams, Tim S. Olds

**Affiliations:** 1 Health and Use of Time Group, Sansom Institute for Health Research, University of South Australia, Adelaide, South Australia, Australia; 2 Respiratory Clinical Research Unit, Repatriation General Hospital, Daw Park, South Australia, Australia; 3 School of Population Health, University of South Australia, Adelaide, South Australia, Australia; 4 Centre for Nutritional Physiology, University of South Australia, Adelaide, South Australia, Australia; 5 Health and Use of Time Group, Sansom Institute for Health Research, University of South Australia, Adelaide, South Australia, Australia; Pulmonary Research Institute at LungClinic Grosshansdorf, United States of America

## Abstract

**Objective:**

To determine the reliability and validity of the Multimedia Activity Recall for Children and Adults (MARCA) in people with chronic obstructive pulmonary disease (COPD).

**Design:**

People with COPD and their carers completed the Multimedia Activity Recall for Children and Adults (MARCA) for four, 24-hour periods (including test-retest of 2 days) while wearing a triaxial accelerometer (Actigraph GT3X+®), a multi-sensor armband (Sensewear Pro3®) and a pedometer (New Lifestyles 1000®).

**Setting:**

Self reported activity recalls (MARCA) and objective activity monitoring (Accelerometry) were recorded under free-living conditions.

**Participants:**

24 couples were included in the analysis (COPD; age 74.4±7.9 yrs, FEV_1_ 54±13% Carer; age 69.6±10.9 yrs, FEV_1_ 99±24%).

**Interventions:**

Not applicable.

**Main Outcome Measure(s):**

Test-retest reliability was compared for MARCA activity domains and different energy expenditure zones. Validity was assessed between MARCA-derived physical activity level (in metabolic equivalent of task (MET) per minute), duration of moderate to vigorous physical activity (min) and related data from the objective measurement devices. Analysis included intra-class correlation coefficients (ICC), Bland-Altman analyses, paired t-tests (p) and Spearman's rank correlation coefficients (r_s_).

**Results:**

Reliability between occasions of recall for all activity domains was uniformly high, with test-retest correlations consistently >0.9. Validity correlations were moderate to strong (r_s_ = 0.43–0.80) across all comparisons. The MARCA yields comparable PAL estimates and slightly higher moderate to vigorous physical activity (MVPA) estimates.

**Conclusion:**

In older adults with chronic illness, the MARCA is a valid and reliable tool for capturing not only the time and energy expenditure associated with physical and sedentary activities but also information on the types of activities.

## Introduction

How we use our time, that is the activities we engage in, can lead to health benefits. Physical activity questionnaires and activity monitors quantify activity durations or movements, from which estimates of energy expenditure can be made and distinctions between differing lifestyles can be inferred. [1] Lifestyles are often classified as “sufficiently active” [meet or exceed recommendations for daily moderate to vigorous physical activity (MVPA)], “insufficiently active” (below recommendations for daily MVPA). The amount of sedentary time (time spent in very low energy expending activities) within either lifestyle can be quantified as either appropriate or excessive. Strong associations exist between time spent in physical activity [2] and sedentary behaviours [3] and physical and/or psychological health outcomes. In chronic obstructive pulmonary disease (COPD), accelerometry data provide evidence of reduced physical activity levels [2], [4]–[7] and disturbed sleep [8].

While activity monitors/sensors can quantify movement and give us estimates of energy expenditure, they are unable to tell us what people are doing. Understanding how people use their time and the time they spend in different kinds of activities or behaviours, is increasingly importantly as evidence indicates that not only are lifestyles linked to health benefits, but the specific activities (i.e. sleep [9], social interaction [10]–[12] and cognitive activities [13]–[14]) within those lifestyles are also linked to health benefits independent of the energy expenditure associated with the activity. For example, watching television and reading, two activities difficult to differentiate based on energy expenditure, have differing impacts on eating behaviours and cognitive load as a result influencing health in different ways. Many associations we are aware of are found within the sedentary and/or inactive lifestyles and, as people with COPD have been reported to have reduced activity profiles, [2], [4]–[7] distinguishing between individual activities is likely to be important. Use of time recalls, unlike physical activity questionnaires or activity monitors, provide researchers with detailed profiles of daily time use. They document every activity a person engages in and obtain important contextual information about specific types and patterns of activities. Despite their use in healthy adolescents [15] and adults [16], these assessments have rarely been used in populations with chronic illnesses. One promising use of time instrument is the Multimedia Activity Recall for Children and Adults (MARCA), which has had test-retest reliability assessed and has been validated against multiple methods in children, adolescents and healthy adults. [17]–[20] In each case the MARCA shows high test-retest reliability and good convergent and criterion validity. Significant differences between the time use patterns observed in youth and adulthood and those observed in people with COPD are likely, in part because people with COPD are generally older but also because this population may; not be in full time employment; have less structured days; and may have varying degrees of cognitive impairment.

This study's aim was to quantify the reliability and validity of the MARCA when compared with objective activity monitoring in people with COPD and their spousal carers.

## Methods

### Participants

A convenient sample of people with a clinical diagnosis of COPD and their spousal carers were recruited from Repatriation General Hospitals' (RGH) clinical and research databases. Ethical approvals were obtained from the University of South Australia (0000024007) and the Southern Adelaide Clinical Human Research Ethics Committee's (054/10). Each participant was informed about the nature and purpose of the study before providing written consent.

### Measurements

During an orientation visit to the RGH respiratory laboratory, all participants completed post-bronchodilator spirometry to confirm the diagnosis and severity of COPD. Each participant also completed the Mini Mental State Examination (MMSE [21]) to ensure appropriate cognitive function (MMSE >25).

Choosing the most appropriate activity-monitoring tool is often difficult. Many devices are available, each recording activity, some however are designed to record different aspects of time use (i.e. SenseWear Pro3® reports sleep). Few however have been validated in people with COPD and reports of agreement between monitors are varied. [22], [23] As a result, participants in this study wore New Lifestyles 1000 (NL-1000) pedometers (*New Lifestyles, Inc., Lee*'*s Summit, Missouri, USA*), Actigraph GT3X+ accelerometers *(Actigraph, Pensacola, Florida USA)* and Sensewear Pro3® armbands *(Body Media, Pittsburgh USA)*. Actigraph GT3X+® and Sensewear Pro3 armbands are widely used in activity monitoring research. They provide modest to good agreement with energy expenditure estimates obtained from doubly labelled water [Actigraph GT3X+® (r = 0.30) [24], Sensewear Pro3® (r = 0.68) [25]] and strong agreement with energy expenditure derived from indirect calorimetry in people with COPD [Actigraph GT3x+® (r≥0.77), Sensewear Pro3® (r≥0.65)]. [23]. A waist mounted pedometer was included to account for potential underestimation of step counts by the Sensewear Pro3® armbands.

Each device was worn for at least six consecutive days and provided measures of physical activity. Their operation and validity have been previously reported (Actigraph GT3X+ [26], [27], Sensewear Pro3® armband [4], [28], [29], NL-1000 pedometer [30]). The Actigraph GT3X+ accelerometer and the NL-1000 pedometer are worn at the hip while the Sensewear Pro3® armband is worn on the bicep. Participants were asked to wear these devices at all times except when undertaking water-based activities, and to maintain a log of their non-wear periods. Each accelerometer recorded activity in 60 second epochs. Wear compliance was set at a minimum 12 hours of valid accelerometry data, allowing for approximately 75% coverage of waking hours. These compliance limits have been previously shown to provide 88% wear compliance. [31] Comparable wear compliance was expected for pedometry. Device “non-wear” was determined using the SenseWear Pro3® data.

During the monitoring period, two MARCA interviews each covering 2 days were undertaken resulting in four separate days of time use data (MARCA interview 1 collected yesterday (day 1) and the day before yesterday (Day 2) data, while MARCA interview 2 collected yesterday (Day 3) and day before yesterday (day 4). Each MARCA interview required around 45 minutes. One of the MARCA interviews was repeated a second time (separated by a minimum of four hours) to assess test-retest reliability.

**Figure 1:**
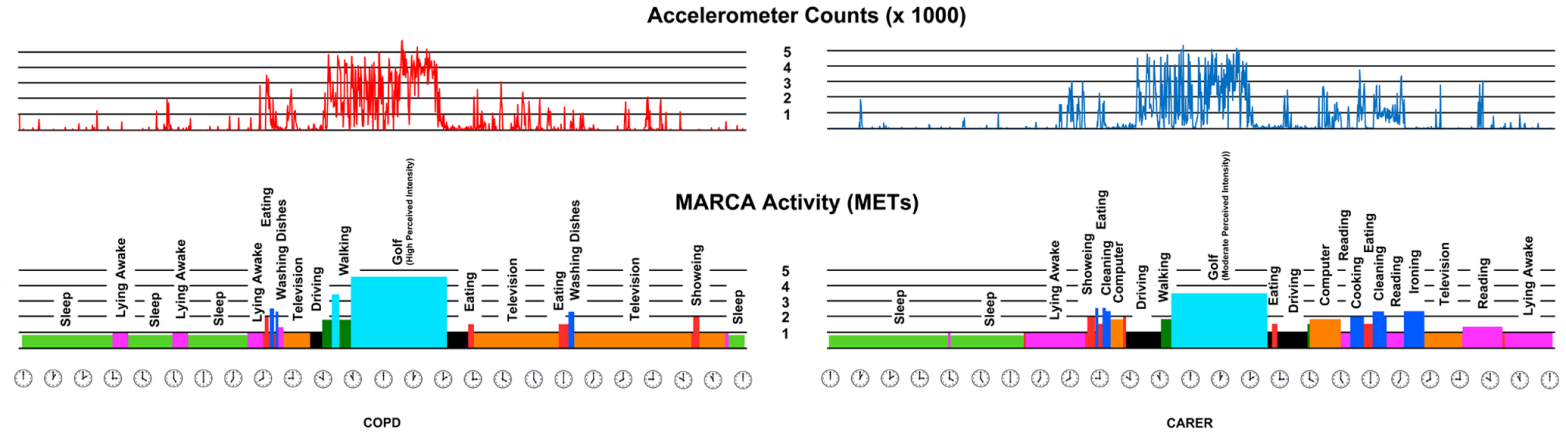
24-hour activity profile for one enrolled dyad.

The MARCA is computer based use of time instrument that, through a structured interview format, records and construct detailed daily activity profiles. [18], [20] Using anchor points such as waking from sleep, breakfast, lunch, dinner and bedtime, the respondent is invited to systematically recall every activity between these anchor points to a resolution of five minutes. Each specific activity, their duration and, where appropriate, their intensities are recorded using drop down boxes. Age specific activity libraries (collapsible into 10 superdomains) consisting of over 500 distinct activities, are embedded within the MARCA permitting automatic assignment of energy expenditure values (in metabolic equivalent of task or METs) to each of the individual activities. The adult library uses the energy estimates published by Ainsworth et al. [32], [33] Figure 1 illustrates the how the MARCA constructs time use profiles and provides time use data at both macro and micro levels.

A recall period consists of two 24-hour days (midnight to midnight), immediately preceding a MARCA interview. Individual recalls can be used as point in time assessments, while sequential recalls can be used to track longitudinal changes to patterns of time use. Strong test-retest reliability (ICC 0.920–0.997) has been documented in the general adult population [18], for the major activity sets; sleep, physical activity levels (PAL) and screen time. Validation studies comparing MARCA derived TDEE with doubly-labelled water [17] and MARCA derived PAL with accelerometer counts/minute [18] show strong correlations (r_s_ = 0.70, r_s_ 0.72) in adolescents and adults respectively.

### Data management and analysis

For the reliability analysis, the average time devoted to groups of activities during both recall days (MARCA superdomain*s*) was calculated. Superdomains are exhaustive and mutually exclusive activity sets. Table 1 lists the superdomains and provides example activities.

**Table 1:** Summary of the 10 Multimedia Activity Recall in Children and Adults (MARCA) superdomains.

	Superdomain	Description	Examples
1	Sport/Exercise	Sport and exercise	Golf, Tennis
2	Screen Time	Television, computers and videogames	Watching TV, Internet
3	Transport	Active locomotion and Passive locomotion	Walking, Driving a car
4	Quiet Time	Time spent without interaction	Reading, Listening to music
5	Self-care	Eating and grooming	Having dinner, Showering
6	Cultural	Arts and crafts	Playing the piano, Painting
7	Work/Study	Occupational activity and study	Clerical work, Homework
8	Chores	Indoor and outdoor household chores	Gardening, Food preparation
9	Social	Interacting in social contexts	Playing cards, Family get-togethers
10	Sleep	All sleep including naps	

In addition, the amount of time spent in; Sedentary (1–1.9 METs); Light (2–2.9 METs); Moderate (3–5.9 METs); and Vigorous (≥6 METs) energy expenditure zones (based on the MARCA's energy expenditure compendium) was calculated. Finally, the total amount of sitting time, and PAL (METs) were calculated.

For the validity analysis, only activity monitoring data corresponding to the four recall days collected via the MARCA interview were used. PAL was calculated from the MARCA using factorial methodology (i.e. the energy cost of a given activity was multiplied by the amount of time reported in each activity, summed across the day, and divided by 1440 minutes). Results presented in this manuscript represent the average of the four day recall period. Moderate to vigorous physical activity (MVPA) was calculated as the total amount of time spent in activities expected to require ≥3 METs. Sensewear Pro3® estimates of MVPA, step counts and accelerometer counts were calculated using proprietary algorithms (*Sensewear Professional 6.1* software). Sensewear Pro3® PAL estimates were converted (Sensewear Pro3® TDEE divided by estimated resting metabolic rate (RMR)) using COPD-specific equations [34] and World Health Organisation (WHO) equations for carers. [35] The total daily step count was recorded from the NL-1000 pedometers, and Actigraph GT3X+ total daily counts using *Actilife 5.5* software. For the NL-1000, Sensewear Pro3® and Actigraph GT3X+, reported values were averaged across the four days coinciding with the MARCA interviews.

Intra-class correlation coefficients and Bland-Altman analysis [36] were used to quantify test-retest reliability and validity when commensurable units were reported (i.e. MARCA PAL vs. Sensewear Pro3® PAL, MARCA MVPA vs. Actigraph GT3X+ MVPA and Sensewear Pro3® MVPA). Paired t-tests were used to probe for significant differences between recall episodes (reliability) and test and reference measures (validity). Spearman's rho correlations were used to quantify validity when units were not commensurable (i.e. MARCA PAL vs. Actigraph GT3X+ counts and MARCA MVPA vs. pedometers steps). MARCA-derived PAL were compared with total Actigraph GT3X+ counts and Sensewear Pro3® corrected PAL, and MARCA-derived MVPA with estimated minutes of MVPA derived from accelerometer counts using the Freedson [37] equation, Sensewear Pro3®-reported MVPA, and total daily steps. All analyses were performed on the whole sample and on the designated dyad members (COPD or carer).

A sample size of 40, assuming real correlations between MARCA PAL and total Actigraph GT3X+ counts of 0.72 [18], would in 95% of cases yield correlations of 0.53–0.84. When split by COPD and Carers (n = 20 per group), correlations between 0.41 and 0.88 were expected. Modest over recruitment was undertaken to account for missing data. No *a priori* criteria for reliability and validity were imposed because we considered reliability and validity outcomes to be continuous rather than dichotomous in nature.

## Results

**Figure 2:**
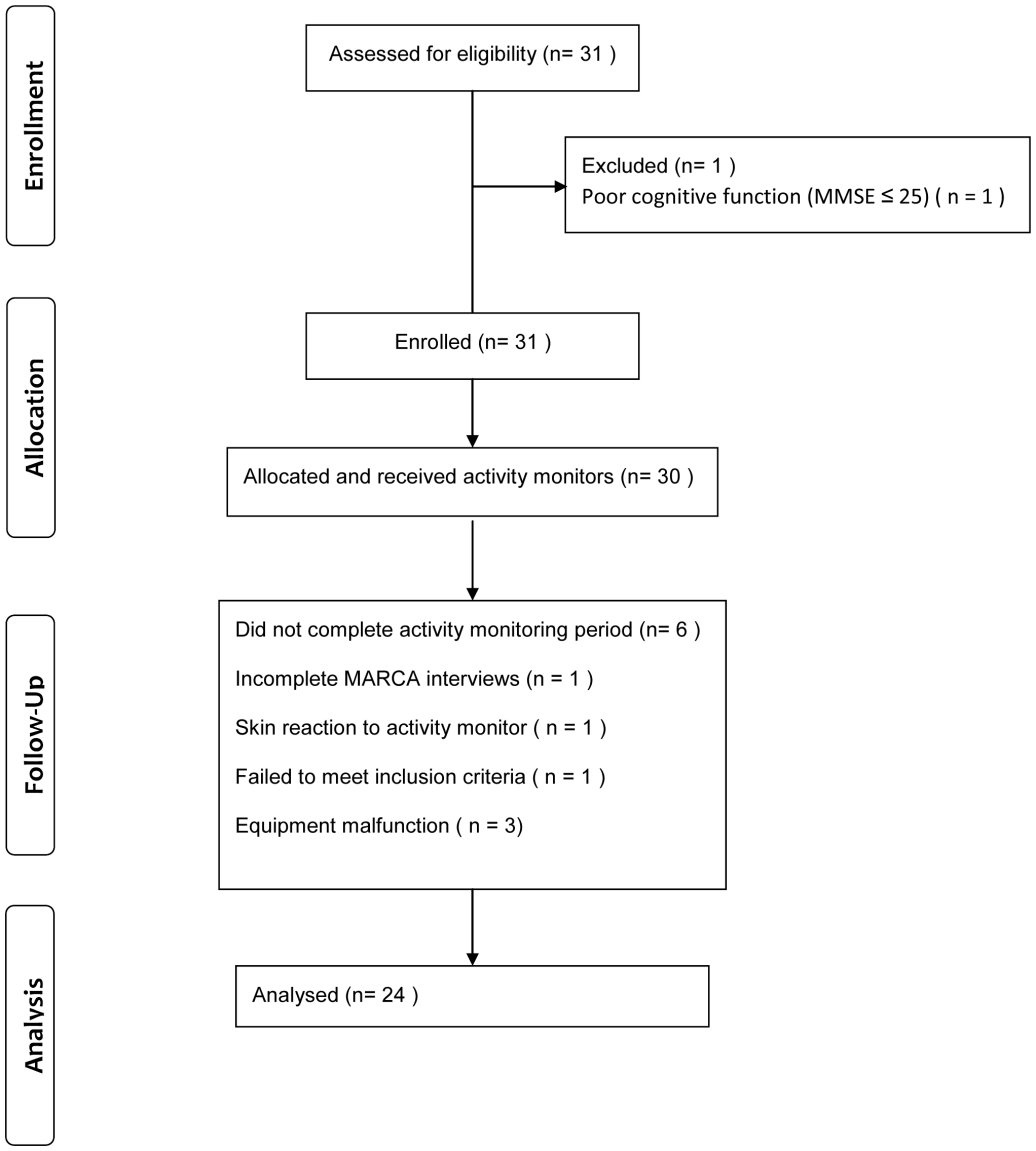
Consort diagram detailing participant flow.

A consort diagram is for this study is shown in Figure 2. Thirty couples agreed to participate and were provided with activity monitors, one couple withdrew (skin reaction from monitoring devices), one couple provided incomplete MARCA interview data, one couple did not meet spirometric COPD criteria and equipment malfunctioned for three couples, leaving a final sample of 24 couples (total n = 48). Participant characteristics are shown in Table 2.

**Table 2:** Baseline demographic data for people with COPD and their carers (n = 24 pairs).

Characteristic		All (n = 48)	COPD (n = 24)	Carer (n = 24)
Age		72.0 (9.7)	74.4 (7.9)	69.6 (10.9)
% Female (n)		50 (n = 24)	25 (n = 6)	75 (n = 18)
BMI (kg.m^−2^)		27.5 (4.1)	27.6 (4.3)	27.5 (4.0)
FEV_1_ (% predicted)		76 (32)	54 (13)	99 (24)
FEV_1_/FVC (ratio)		61.7 (17.9)	47.8 (12.9)	75.6 (9.2)
GOLD stage (n)	0	19	0	19
	I	6	5	1
	II	10	7	3
	III	13	12	1
	IV	0	0	0
MMSE		28 (1)	28 (2)	28 (1)

All values are expressed as Mean (SD). BMI: body mass index, FEV_1_: forced expiratory volume in 1 second, FEV_1_/FVC ratio: Forced expiratory volume in 1 second (FEV_1_) expressed as a percentage of Forced vital capacity (FVC), GOLD: global initiative for obstructive lung disease stage, MMSE: Mini Mental State Examination.

### Test-retest reliability

**Figure 3:**
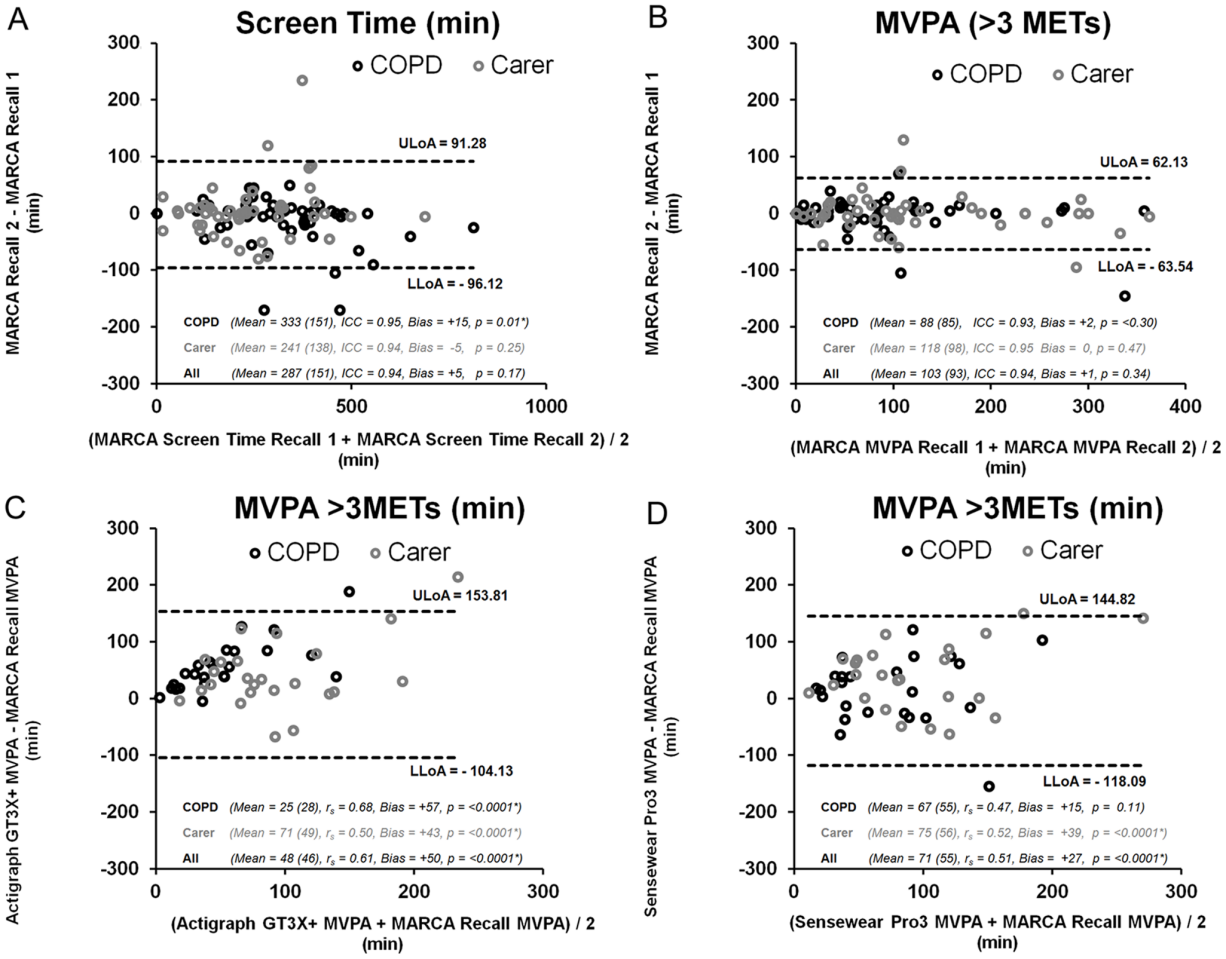
Bland-Altman plots for selected reliability and validity outcomes. Test-retest reliability of the MARCA superdomains MVPA and Screen Time are shown in the top two panels (MVPA - panel A, Screen Time - panel B). Validity comparisons between MVPA estimates from Actigraph GT3X+ and Sensewear Pro3® activity monitors and MARCA MVPA estimates are shown in the bottom two panels (Actigraph GT3X+ MVPA vs. MARCA MVP - panel C, Sensewear Pro3 MVPA vs. MARCA MVPA - panel D). COPD: Chronic obstructive pulmonary disease, MET: Metabolic equivalent, r_s_: Spearman's rank correlation coefficient, ICC: Intra-class correlation coefficient.

Test-rest reliability outcomes for the 10 MARCA superdomains, the energy expenditure zones and total sitting time are presented in Table 3. Bland-Altman plots show good agreement and small biases between recalls for Screen Time (Figure 3, panel A) and MVPA (Figure 3, panel B) superdomains.

**Table 3:** Test-retest reliability of the selected MARCA superdomains, total sitting time, and energy expenditure zones.

Domain	Participants	Mean (SD)	ICC	Bias	Limits of Agreement	T-test (P)
Sports/Exercise (min/d)	All	10 (32)	0.99	0	−10 to +10	0.50
	COPD	8 (33)	0.99	0	−10 to +9	0.39
	Carers	11 (31)	0.99	0	−9 to +10	0.39
Screen Time (min/d)	All	287 (151)	0.94	+5	−91 to +101	0.17
	COPD	333 (151)	0.95	+15	−70 to +164	0.01*
	Carers	241 (138)	0.94	−5	−104 to +94	0.25
Sleep (min/d)	All	489 (115)	0.92	−7	−83 to +98	0.07
	COPD	485 (126)	0.90	+10	−103 to +123	0.11
	Carers	492 (103)	0.96	+4	−57 to +66	0.19
Total sitting time (min/d)	All	666 (142)	0.89	−10	−139 to +119	0.07
	COPD	718 (134)	0.87	−10	−147 to +127	0.16
	Carers	614 (131)	0.89	−10	−132 to +111	0.13
Sedentary (1–1.9 METs) (min/d)	All	662 (147)	0.90	−7	−142 to +129	0.16
	COPD	717 (136)	0.86	−7	−149 to +136	0.27
	Carers	607 (137)	0.88	−8	−137 to +122	0.22
Light (2–2.9 METs) (min/d)	All	184 (116)	0.94	−2	−79 to +76	0.34
	COPD	148 (81)	0.86	−6	−91 to +79	0.18
	Carers	221 (133)	0.97	+2	−67 to +72	0.32
MVPA (≥3 METs) (min/d)	All	103 (93)	0.94	+1	−62 to +65	0.34
	COPD	88 (85)	0.93	+2	−61 to +66	0.30
	Carers	118 (98)	0.95	0	−64 to +65	0.47
TDEE (MET.min)	All	2029 (302)	0.96	−13	−188 to +163	0.08
	COPD	1948 (291)	0.95	−16	−196 to +164	0.11
	Carers	2110 (291)	0.96	−9	−181 to +163	0.23

ICC: intra-class correlation coefficient, MET(s): metabolic equivalent(s), MVPA: moderate to vigorous physical activity, TDEE: total daily energy expenditure. *: indicates statistically significant results.

Reliability was uniformly high, with coefficients for all but cultural activities and self-care greater than 0.85. In all domains except screen time, reliability coefficients were slightly higher for carers than for their partners with COPD. Biases were small and with the exception of social activities (p<0.03) and screen time in people with COPD (p<0.02) no significant test-retest recall differences existed. For all major health-related categories of time use [TDEE (ICC = 0.95–0.96), MVPA (ICC = 0.93–0.95), sleep (ICC = 0.90–0.96), sitting time (ICC = 0.87–0.89) and screen time (ICC = 0.94–0.95)] reliability was high. Reliability of the MARCA was unaffected by the day recalled (yesterday or the day before yesterday) with ICC's for major health related categories of time use consistently above 0.88.

### Validity

All participants had acceptable wear compliance with average effective wear time exceeding 23 hours for each monitor during activity recall periods. Validity data comparing MARCA-derived PAL and MVPA to other assessment methods are presented in Table 4. Bland-Altman plots show good convergent validity with small biases between Actigraph GT3X+® MVPA and MARCA MVPA estimates (Figure 3, panel C) and Sensewear Pro3® MVPA and MARCA MVPA estimates (Figure 3, panel D). Validity correlations were moderate to strong (MVPA r_s_ = 0.43–0.80; PAL r_s_ = 0.43–0.80). These correlations remained strong when non-wear periods were excluded from analysis (MVPA r_s_ = 0.43–0.80; PAL r_s_ = 0.46–0.70) and minimal differences were observed when data was analysed as individual days (MVPA r_s_ = 0.43–0.80; PAL r_s_ = 0.43–0.80). MARCA-derived MVPA yielded significantly higher estimates when compared to either activity monitor.

**Table 4:** Validity comparisons between MARCA-derived and objective outcome measures.

MARCA measure	Population	Mean (SD)	Reference measure	Population	Mean (SD)	r_s_	ICC	Bias	Limits of Agreement	T-test (P)
PAL (METs)	All	1.41 (0.16)	Actigraph GT3X+ counts	All	431722 (2113383)	0.69	NA	NA	NA	NA
	COPD	1.35 (0.14)		COPD	318273 (146389)	0.74	NA	NA	NA	NA
	Carers	1.47 (0.16)		Carers	545171 (207409)	0.56	NA	NA	NA	NA
			Sensewear Pro3® PAL, (METs)	All	1.33 (0.26)	0.65	0.56	+0.08	−0.31 to +0.47	0.002
				COPD ^§^	1.48 (0.23)	0.66	0.33	+0.18	−0.17 to +0.51	<0.0001*
				Carers ^†^	1.17 (0.19)	0.50	0.62	−0.01	−0.36 to +0.34	0.39
MVPA (min)	All	97 (66)	Actigraph MVPA (min)	All	48 (46)	0.61	0.41	+50	−54 to +154	<0.0001*
	COPD	81 (57)		COPD	25 (28)	0.68	0.30	+57	−29 to +142	<0.0001*
	Carers	114 (72)		Carers	71 (49)	0.50	0.41	+43	−77 to +163	<0.0001*
			Sensewear Pro3® MVPA (min)	All	71 (55)	0.51	0.47	+27	−91 to +145	<0.0001*
				COPD	67 (55)	0.47	0.42	+15	−102 to +132	0.11
				Carers	75 (56)	0.52	0.49	+39	−78 to +156	<0.0001*
			NL-1000 steps	All	18995 (12913)	0.66	NA	NA	NA	NA
				COPD	12992 (9803)	0.80	NA	NA	NA	NA
				Carers	24999 (13016)	0.43	NA	NA	NA	NA

ICC: intra-class correlation coefficient, PAL: physical activity level, COPD: chronic obstructive pulmonary disease, MET(s): metabolic equivalent(s), MVPA: moderate to vigorous physical activity, Min; minutes, NA: not applicable; *: indicates statistically significant results; **^§^**: Converted using COPD specific RMR equations; ^†^: Converted using WHO height and weight equations.

## Discussion

This study documents the reliability and validity of a use of time instrument that has not been previously used in this population. A fundamental difference between the MARCA and other monitoring methods is its ability to provide estimates of activity not only at a macro level (PAL and MVPA) but also detailed information at a micro level about what types of activities a respondent undertakes. We report excellent test-retest reliability and good convergent validity of the MARCA when compared to the Actigraph GT3X+ accelerometer, Sensewear Pro3® armband, and the NL-1000 pedometer. Our data show moderate to strong agreement between the MARCA and both the Actigraph GT3X+ and Sensewear Pro3® (r_s_ = 0.50–0.74 for PAL; r_s_ = 0.47–0.68 for MVPA) (Table 4). Results for both people with COPD and their carers were strikingly similar to those determined on younger, healthy adults. [18]

Criterion validation tools were either cost prohibitive (e.g. doubly labelled water) or considered too intrusive (e.g. video recording) for validity comparisons in this study. We instead measured convergent validity against “measured” counts of activity (Actigraph GT3X+ counts) and “calculated” activity outcomes (PAL and MVPA), based on the MARCA's inbuilt energy expenditure compendium. Using calculated outcomes may be considered a limitation, however as most activity monitoring research report activity in this way due to its immediate intuitiveness; our outcomes also included converted data. Table 4 shows little difference between validity correlations of “measured” (GT3X+® PAL r_s_ = 0.56–0.74) or “calculated” outcomes (SenseWear Pro3® PAL r_s_ = 0.49–0.66).

Understanding how days are constructed is important on multiple levels. Growing evidence confirms that independent of energy expenditure, specific activity domains as well as the context in which they are undertaken influence health outcomes. The physical and psychological benefits of activity vary by domain [38], [39] and specific types of activity (e.g. cognitively demanding leisure activities [14]), their degree of social interactions [11], [12] and their enjoyment levels [40], [41]) can affect health in older adults. The MARCA has the potential to provide contextual information that improves our understanding of activity patterns. In turn allowing targeted interventions aimed at modifying behaviour in an attempt to reduce disease burden (i.e. promotion of movement during television commercials during extended bouts of television viewing) to be designed and implemented.

A six day monitoring protocol was chosen as differences in the types of activities undertaken on weekend and week days within a week are likely to occur. Activity monitoring data was only used to validate the four days of activity recall and in order to facilitate mutually agreeable MARCA interview times, two additional days of monitoring were included. Participants were asked to include at least one weekend and one week day during their activity recall periods. By assessing reliability in this way, it was evident that people with COPD and their carers were able to adequately recall activities undertaken during the preceding two days, and where present, the recall biases were small (< 15 min). Because diurnal variations in time use (across and within time use domains) and dose-response relationships (between domains of time use and health outcomes) have as yet, not been quantified in people with COPD, the clinical significance of the observed biases is unknown. We believe it is extremely unlikely these biases to be of significance due to their relatively small sizes.

Assessment using a dyad (COPD and carer) was novel. It allowed for validity to be assessed on two similarly aged (COPD 74.4±7.9; Carer 69.6±10.9), cohabitating members of a dyad each with distinct levels of capacity. Figure 1, shows the 24-hour activity pattern of one enrolled dyad. Despite similar macro level movement patterns (accelerometry counts), there were significant differences in the types of activities undertaken at the micro level of time between the two dyad members. Additional analyses are planned for this and other similar use of time data to better understand activity patterns in people with chronic health conditions but were omitted from this manuscript as it's focus is assessing the MARCA's reliability and validity.

MARCA interviews were very well tolerated. Only one research dyad failed to provide complete MARCA interview data in our study. The majority of participants enjoyed being interviewed. The MARCA analytical software and “one-click” analysis mean the MARCA has low researcher burden.

Unlike standard physical activity questionnaires, the MARCA, does not collect answers to broad statements (e.g. “How many minutes did you spend walking yesterday?”) but rather reconstructs entire days. As a previous day physical activity recall, the MARCA relies on respondents being able to recall detailed accounts of a given day. Two separate days are unlikely to be constructed identically and recall precision will deteriorate with time. Assessments of the test-retest reliability of previous day activity recalls are as a result required to be undertaken on the same calendar day [42].

This study was not without limitations. Firstly a relatively small sample size was used that was biased towards participants with severe COPD. Nearly fifty percent of enrolled participants with COPD had a diagnosis of severe COPD according to GOLD guidelines. [43] As the aim of this study was to confirm the reliability and validity of the MARCA in people with COPD we did not include functional limitation measures (i.e. six minute walk tests or Medical Research Council scales for respiratory related impairment) which means we are unable to provide comment on how well our research sample represents broader groups of people with COPD with differing levels of functional limitations. We also infer the observed activity profiles are due to COPD, however many within aged populations have chronic comorbid conditions that independently modify time use. Multiple self-reported comorbidities were common in our participants, with cardiovascular disease and musculoskeletal conditions being the most common. Spirometric assessment revealed twenty percent (n = 5) of carers met GOLD criteria for a COPD diagnosis despite not identifying themselves as a person who had COPD. When participants were allocated to either a COPD or non-COPD group based on spirometric criteria, there was little change in the reliability (ICC = 0.53–1.00) and validity correlations (r_s_ = 0.26–0.77).

Potential misreporting of activity by participants may have also occurred due to; unnoticed movements, inaccurate recall or perceived social expectations. The inclusion of objective monitoring devices was designed to assess this potential limitation, with validity correlations between the devices showing bias of this nature unlikely. Comparisons of validity required correct device wear. Participants were instructed on the use of each device, and were provided with instructions for each device. In addition participants were asked to keep a non-wear diary. Device wear was estimated using SenseWear Pro3® data (assuming all devices were worn simultaneously), by extracting self report non-wear and by applying algorithms designed to identify bouts of consecutive zero counts lasting more than 90 minutes in accelerometry files. Using these approaches, activity recall periods had between 97% and 99% coverage with activity monitoring devices, Simultaneous device wear was assumed and Sensewear Pro3® data was used to determine non-wear. Validity results remained strong even when these non-wear periods were removed from analyses.

One further consideration is that consistent with most recall instruments improved reliability and validity outcomes were observed in our sample than in previous adolescent reports. Despite initial development in adolescents, each of the current activity libraries contain both universal and specific age related activities. However, the majority of energy expenditure values have been derived from the younger adult population. The modest differences in reliability/validity analyses between our sample and previous studies in adolescence may simply be a function of the different age specific nature of activities.

MARCA derived estimates of MVPA (98+/−66 mins) were significantly (p<0.0001) higher than Actigraph GT3X+® (47+/−46 mins) or Sensewear Pro3® estimates (71+/−55 mins). These observations were in part likely due to differences in how the MARCA captures use of time data. People recall individual activities, associated durations and report the activities average intensity (e.g. playing moderate intensity tennis for an hour), while activity monitors report actual movement. In an hour's tennis, for example, where the average intensity exceeds the MVPA threshold, there may be many inactive periods. It is also possible that the current energy expenditure compendium, based largely on data from young adults, does not accurately capture the true energy costs experienced by people with COPD or their carers. This may also be the reason behind the observed differences between MARCA derived PAL (1.40+/−0.17) and PAL values obtained from the SenseWear Pro3® (1.33+/−0.26). Older people (with or without COPD) may expend greater energy performing similar tasks due to decreased mobility, dyspnoea or underlying chronic disease processes, resulting in a greater perceived effort. Alternatively, activities may be undertaken more slowly with lower energy expenditure, resulting in inconsistencies between the MARCA calculated and accelerometer recorded energy costs.

People with COPD had marginally weaker correlations compared to their nominated carer. Secondary analysis undertaken after grouping participants according to whether or not they met spirometric diagnosis of COPD, also found marginally weaker correlations in people with spirometric confirmed COPD. Decreased cognitive ability and potentially greater variance between individual daily routines within COPD populations may account for these observations. Despite this, similar patterns of activity were observed in both groups, with the only minor difference being within the superdomain “chores”, where carers spent a greater proportion of time. Whether this was resultant from a reduced capacity experienced by those with COPD or because of historic divisions of domestic labor according to gender is unknown.

### Implications

Activity promotion is a primary management strategy for people with COPD. [41] The MARCA permits recording of individual and potentially group use of time profiles, allowing identification of periods where potentially detrimental health behaviours regularly occur, raising the possibility of designing specific behavioural strategies. Growing evidence supports the link between adverse health outcomes notably cardio metabolic disorders [44], [45], hypertension [46] and insulin resistance [47], [48] and sedentary behavioural patterns. These conditions are among the more common comorbidities in people with COPD [49], [50], so altering behaviour to reduce sedentary activities should be of importance in this population.

Accelerometer recordings are recognized as one of the most effective estimates of macro level habitual physical activity [51], despite this, use in people with COPD remains limited due to their cost and lack of validation studies. [22] The relationship between functional clinical assessments (walk tests etc.) and habitual daily activity has been explored, with conflicting views on their usefulness. [2], [52], [53] Each individual monitor collects and interprets data differently meaning predictive equations were required to convert the Actigraph GT3X+ counts [35] and Sensewear Pro3® PAL [34], [35] into outcomes that are comparable across devices. As a computer assisted phone-based interview, the MARCA, affords researchers simplicity and flexibility when designing research protocols but perhaps of greatest importance, commonality in a wide variety of activity and use of time outcomes.

## Conclusion

Given the increasing body of evidence linking adverse health outcomes with specific activity types, understanding of how people with COPD use their time on a micro level is important for both researchers and clinicians. In this study we have confirmed the MARCA as a reliable, valid and easy to administer, use of time instrument in people with COPD. By collecting information on a micro level, the MARCA is able to provide high-resolution snapshots of the types of activities routinely undertaken by people with COPD during a 24-hour recall period. The MARCA provides researchers with either a standalone or complementary assessment tool when exploring habitual activity in COPD populations.
